# Returning to employment following allogeneic hematopoietic stem cell transplant: A major problem among survivors

**DOI:** 10.1002/jha2.788

**Published:** 2023-10-09

**Authors:** Luis Filgueira, Amir Steinberg, Rochelle Mendonca, Seah H. Lim

**Affiliations:** ^1^ Programs in Occupational Therapy Columbia University Irving Medical Center New York City New York USA; ^2^ Department of Medicine Division of Hematology and Oncology New York Medical College Valhalla New York USA; ^3^ Department of Medicine Division of Hematology and Oncology State University of New York Upstate Medical University Syracuse New York USA

**Keywords:** allogeneic transplant, chronic complications, multidisciplinary occupation‐focused approaches, returning to employment

## Abstract

Quality of life (QoL) is an important aspect of cancer survivorship. One of the most acute problems that impact survivors in many aspects of activities of daily living and compromise their QoL is the inability to return to employment following successful cancer therapy. This is most prominent among survivors after allogeneic hematopoietic stem cell transplant (allo‐HSCT). More than 50% of the survivors following allo‐HSCT remain unemployed one year after the procedure. This problem extends beyond the initial few years; unemployment rates among those who underwent allo‐HSCT during their childhoods or adolescence have remained high. The inability to return to employment imposes a financial burden. Survivors following allo‐HSCT also experience a multitude of chronic psychosocial complications that may be both contributing and consequential to the inability to return to employment. However, many transplant programs and cancer centers do not have return‐to‐employment programs. In this review paper, we discuss the prevalence of unemployment following allo‐HSCT. We examine the psychosocial symptoms experienced by survivors and how they may affect survivors’ ability to return to employment. Finally, we propose a multi‐disciplinary multi‐pronged occupation‐focused approach to address the complex and inter‐related psychosocial symptoms to help alleviate the problem.

## 1 INTRODUCTION

Advances in modern chemoimmunotherapeutic approaches have provided many adult patients, especially those with hematologic malignancies, with the prospect of long‐term survival. Quality of life (QoL), therefore, becomes an important issue in cancer survivorship. One important dimension in QoL for many survivors is the ability to return to employment [1]. Being employed is often viewed as a measure of self‐worth, competence, and independence. Unfortunately, many cancer survivors face treatment‐related long‐term complications, both physical and psychosocial, that may directly or indirectly affect their ability to return to employment. The problem of inability to return to employment is particularly acute among survivors who have undergone allogeneic hematopoietic stem cell transplant (allo‐HSCT) because allo‐HSCT is not only a complex procedure but also involves prolonged hospitalization and frequent hospital readmissions, especially in the first few months after the procedure.

There is a high prevalence of long‐term complications among survivors of allo‐HSCT. Nearly 90% of transplant centers acknowledged that the inability to return to employment is one of the most serious problems facing survivors [2]. Being able to return to employment is a good indicator of the recovery and functional status of survivors. Not being re‐employed affects many aspects of daily life and QoL. The Person‐Environment‐Occupation (PEO) model proposes that the person, environment, and occupation interact regularly over time and space, which can increase or decrease a person's engagement and participation in life activities [3]. The better the fit or compatibility between person, environment, and occupation, the greater the performance in life occupations. Strategies to alleviate the problem of inability to return to employment should, therefore, be considered from this lens. The ability to return to work depends on whether the survivor is physically and mentally prepared, whether the job held by the survivor is still open, and whether the employer is willing to re‐employ the survivor and provide any necessary adjustment in the work environment to accommodate the survivor's need to accomplish satisfactory employment performance. In this research, we carried out a literature search on the inability to return to employment following allo‐HSCT, in particular, for literature examining the extent of the problem, factors contributing to challenges, and measures that have been attempted to overcome the problems. Based on the results, we appraise the prevalence of inability to return to employment among these survivors. We next discuss the multi‐directional interactions between the inability to return to employment and the psychological/emotional, social, and physical problems faced by the survivors. Finally, we propose a multidisciplinary multi‐pronged occupation‐focused approach that could be used in the functional recovery of these survivors. Our discussion is not only relevant to survivors following allo‐HSCT but also to survivors after intensive chemotherapy for hematologic malignancies who do not require allo‐HSCT.

## 2 PREVALENCE OF INABILITY TO RETURN TO EMPLOYMENT FOLLOWING ALLO‐HSCT

Allo‐HSCT is a complex and highly involved medical procedure. Survivors are at high risk for inability to return to employment because of prolonged inpatient hospital stays and recurrent hospitalizations due to complications. The median initial hospital length of stay is 25 days [4]. Following discharge from the hospital, frequent clinic visits, initially up to three days a week are needed. Hospital readmissions due to complications are also common [5, 6, 7], particularly during the first 100 days after transplant. The most common reason for readmission is opportunistic infections due to immunosuppression, both from impaired immune function after the transplant and from the use of immunosuppressive agents to prevent graft‐versus‐host disease (GVHD). Even though the US Family and Medical Leave Act provides employees with up to 12 weeks of unpaid, job‐protected leave per year, these 12 weeks are often not long enough and place survivors at risk of losing their employment.

Data on the prevalence of inability to return to employment is variable. A study of 105 survivors in France found that, with a median time of 15 months following allo‐HSCT, only 52% of the survivors returned to employment [8]. Female gender (odds ratio 2.9) and age (odds ratio 3.2) predicted higher risks. The high rate of inability to return to work extends beyond the 5‐year time point. A study of 203 long‐term survivors (>5 years) in Switzerland found that only 77% were working full‐time or part‐time [9]. In the US, returning to full‐time employment following HSCT was observed in only 60% of the long‐term survivors and 32% in part‐time employment [10]. Among these patients, only 9% returned to full‐time employment at 6 months, and 36% at one year [10]. There is also a paucity of data to show if the patients returned to their previous employment or alternative, and perhaps, employment of lesser responsibilities. Finally, with a median follow‐up of 8 years, only 52% returned to full‐time employment, 27% part‐time, and 17% were on sick leave, disability pension, or old‐age pension in Sweden [11]. Risk factors identified for unemployment in this study included multi‐morbidity, female genders, higher age at diagnosis, and poorer health. Although the higher risks for unemployment among some of the older survivors may be due to a personal choice to retire early, these studies highlight the long‐term impact of allo‐HSCT on return to pre‐transplant employment status. The variability of data on the prevalence of inability to return to work, especially in the early stage after transplant, most likely reflects the severity and extent of early transplant‐related complications such as infections, preparative regimen‐induced toxicities, and the presence of any acute GVHD. They may also reflect differences in change of perspectives in life among survivors in different communities.

The long‐term impact of allo‐HSCT on returning to employment is further exemplified in survivors who received transplants in their childhood, adolescence, or young adulthood. Here, not only are pre‐transplant employments affected, but full‐time schooling and vocational training, both of which may affect the competitiveness of the survivors for future employment, are also adversely interrupted [12]. In a group of 2844 survivors who underwent allo‐HSCT in childhood, unemployment rates were persistently high at all attained ages: 14% between ages 18 and 22 years, 15% between ages 23 and 27 years, and 13% between ages 28 and 32 years, if they were transplanted in the United States [13], and were even higher among those transplanted outside the United States: 56% between ages 18 and 22 years, 53% between ages 23 and 27 years, and 68% between ages 28 and 32 years [13]. Of those who received their transplant in young adulthood (age 18–39 years), only 50.7% worked full‐time and 10.5% part‐time 3 years after the transplant [14]. It is, however, possible that the unemployment rates here may be over‐estimated since in some of the younger survivors, this might be related to being in schools/colleges.

## 3 RELATIONSHIP BETWEEN RETURNING TO EMPLOYMENT AND PSYCHOSOCIAL AND PHYSICAL COMPLICATIONS

The most immediate effect that the inability to return to employment has on survivors and their families is a lack of income and loss of healthcare insurance since most healthcare insurance is provided by employers in the United States [15]. A study found that 47% of the survivors experienced financial hardship characterized by more than a 50% drop in household income, selling/remortgaging their homes, or withdrawing money from retirement accounts [16]. Three percent of the responders in this study reported a declaration of bankruptcy. The financial hardship due to being unemployed is further exacerbated by the need for copayments and out‐of‐pocket payments for expensive medications, hospital readmissions, and clinic visits. The financial burden will affect treatment adherence due to concerns about costs [16]. Ultimately, financial hardship affects the QoL of the survivors [17].

**FIGURE 1:**
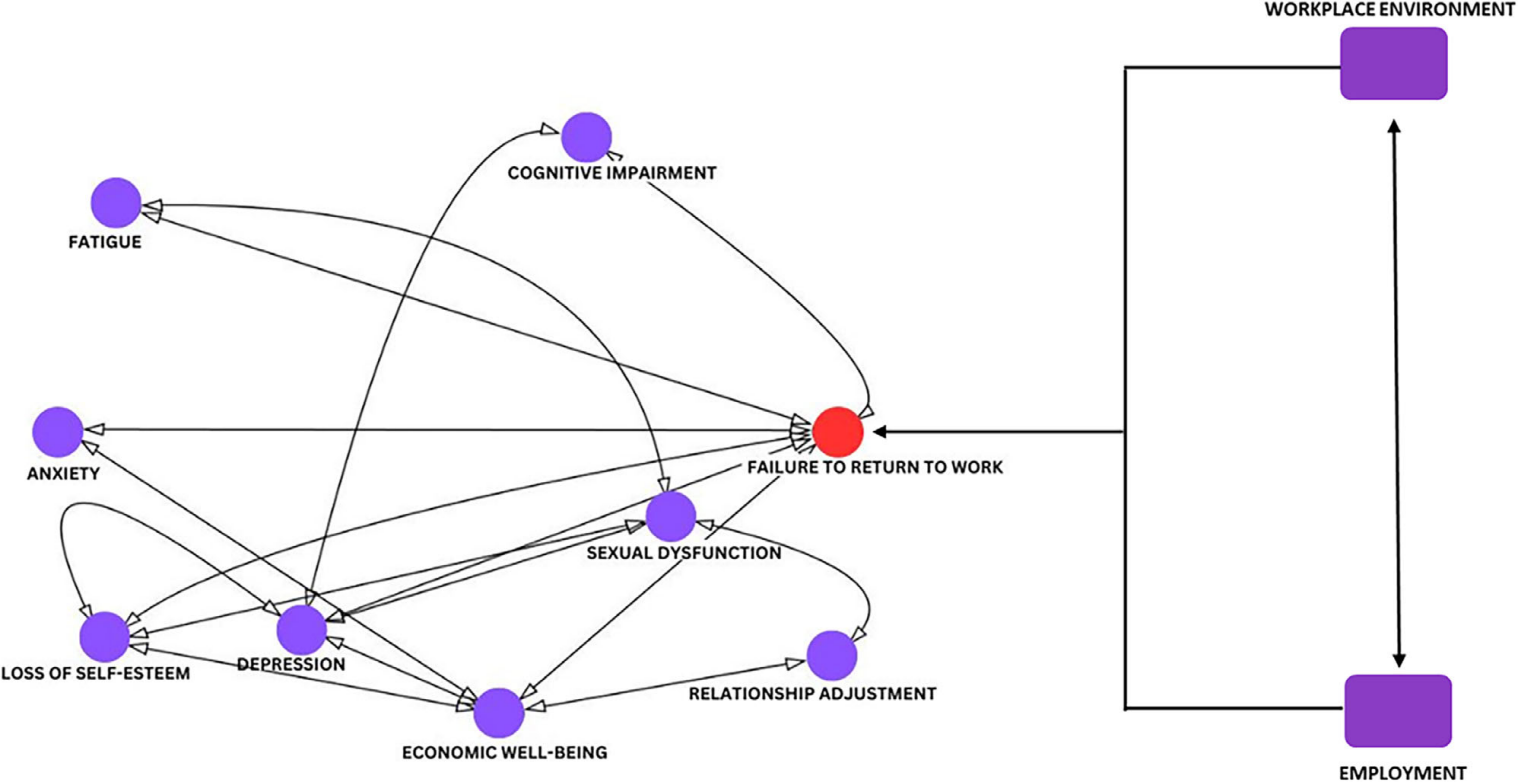
A complex multi‐directional interaction between the personal circumstances experienced by a survivor, his/her environment, occupation demands, and workplace. Successful return to employment and occupational performance relies on addressing these aspects affecting the survivor.

The ability to return to employment is an important dimension of recovery among many survivors following allo‐HSCT. Employment may also provide the survivors with the opportunity and environment for social participation. It improves the social and economic standing of the survivors and their health‐related QoL. It is important to note that the inability to return to employment is both contributing to and consequential to the many chronic psychosocial problems experienced by the survivors. As proposed by the PEO model, although the physical and psychosocial complications faced by the survivors are important determinants, the ability to return to employment is also influenced by the environment and the nature of the work that the survivors do. Environmental factors may be cultural, institutional, social, socio‐economical, or physical, whereas the nature of the work includes consideration of performance, maintenance, expression, and fulfillment. This intersectional relationship between returning to employment, the psychosocial complications experienced by survivors, the environment the survivors are in, and the survivors’ occupation is represented in Figure 1.

The inability to return to employment may potentially change the status of the survivors in the family. It is, therefore, not surprising that survivors who fail to return to employment experience a whole range of secondary psychosocial symptoms (see below), although these symptoms may themselves also be the direct or indirect obstacles to returning to employment. In this section, we discuss the relationship between the chronic psychosocial complications experienced by many survivors following allo‐HSCT and how they may impose direct and indirect obstacles for survivors to return to employment.

### 3.1 Fatigue

Fatigue interferes with daily activities and impacts work performance [18]. Long periods of inactivity related to allo‐HSCT results in weakness, poor exercise tolerance, and decreased muscle strength [19]. Because fatigue reduces concentration [20] and impairs work performance [18], it is a major obstacle to returning to employment. Fatigue is a common symptom among survivors after allo‐HSCT [21]. Symptoms of fatigue occur early in the course of treatment. Fatigue is reported in 90% of the survivors during the first 30 days after transplant [22]. The initial fatigue is induced by the high‐dose chemotherapy and/or radiation that the survivors receive as transplant preparative regimens and is made worse by decreased ambulation and isolation because of severe neutropenia and thrombocytopenia. Other factors contributing to fatigue include electrolyte imbalance from the use of antibiotics and immunosuppressive agents, infections, severe anemia, sleep disorders in the hospital, stress, anxiety, and depression [23]. The prevalence of severe fatigue decreased gradually to 81% by day 100 [24], 41% in the first 5 years [24], and 32% between years 5 and 10 [24]. Unfortunately, the inability to return to employment intensifies the inactivity and may further exacerbate symptoms of fatigue.

### 3.2 Anxiety/Depression

Anxiety/depression and post‐traumatic stress disorder (PTSD) negatively impact occupational performance and the ability to return to employment [25]. Anxiety/depression is a common complaint following allo‐HSCT [26]. It affects survivors across the spectrum of pre‐transplant psychiatric morbidities, including those who have low pre‐transplant levels of depressive symptoms [27]. Anxiety/depression in these survivors includes the worry about the recurrence of the underlying diseases for which the allo‐HSCT is performed and the financial burden due to the procedures. Furthermore, survivors experience isolation imposed on them due to their increased susceptibility to opportunistic infections [28]. Clinically significant depressive symptoms occur in more than 25% of the survivors during the first year after allo‐HSCT [29]. The symptoms may persist for a long time after transplant.

Post‐transplant anxiety/depression may also be associated with PTSD. A study of 206 survivors found clinically significant symptoms of PTSD in 18.9% of survivors during the first six months after transplant [30]. These survivors experienced hypervigilance, avoidance, and intrusion symptoms. Conversely, the financial burden associated with being unemployed precipitates further anxiety/depression, and may lead the survivors into a spiral of deterioration.

### 3.3 Cognitive impairment

Cognitive impairment is a major obstacle to an individual's emotional, physical, and social functioning [23], and consequently QoL. It interferes with an individual's ability to function optimally in employment [31]. Cognitive impairment, in the domains of attention and concentration, memory, executive functioning, and mental processing speed, and its impact on motor functions frequently affects more than 50% of cancer survivors and those following allo‐HSCT [32, 33]. Over 25% of these survivors experienced moderate to severe symptoms [32, 33]. The underlying causes for this complication are not clear but are predominantly treatment‐related, from systemic chemotherapy, long‐term high‐dose corticosteroid use, and cranial irradiation especially in acute lymphoblastic leukemia (ALL) survivors, and high‐dose chemotherapy with or without radiation given to the survivors as part of the transplant preparative regimen. Cognitive impairment resulting from successful treatment of the underlying malignancies, therefore, is a major obstacle for the survivor to return to employment satisfactorily.

### 3.4 Sleep disorders

Sleep disorders exacerbate fatigue and impair an individual's occupational performance. They have been correlated with obstacles to returning to employment [34]. Sleep disorders are common among survivors who have undergone allo‐HSCT [35]. The sleep disruptions include difficulty falling asleep, staying asleep, awakening earlier than intended, and/or non‐restorative sleep. Sleep disruptions affect the QoL of survivors [36, 37]. They also adversely affect the cognition [23]. Despite their prevalence, transplant physicians seldom discuss the complications with the survivors [38].

### 3.5 Relationship adjustment and sexual dissatisfaction

Anxiety/depression negatively impacts occupational performance and the ability to return to employment [25]. Relationship dissatisfaction is often associated with depressive symptoms [39] and may pose an indirect obstacle to satisfactory occupational performance and contribute to the inability to return to employment. Cancer survivors, especially those following allo‐HSCT, commonly experience sexual dissatisfaction and the need for relationship adjustment. Both issues undermine the emotional state and self‐esteem of the survivors and affect the QoL [40]. They also exacerbate depression that interferes with occupational performance [41]. Conversely, sexual dysfunction, especially among males, affects those who are unemployed [42]. Relationship dissatisfaction was reported by partners of survivors, especially of the female gender, 6–12 months following the transplant [43]. Factors contributing to this problem include time required for caregiving, and financial sacrifices as a result of the inability to return to employment by the survivors or interruption to the spouses’ employment and career aspirations [44]. Divorce is less common, being reported in 7% of the cases in the first 5 years after transplant [43]. Relationship dissatisfaction was primarily intrapersonal in female spouses and not with male partners in the first 2 years but became interpersonal, between survivors and spouses 3–5 years after transplant [43].

Sexual dysfunction is reported in nearly 50% of survivors [45]. Documented problems include decreased libido, genital chronic GVHD (cGVHD), hormonal deficiency, erectile dysfunction, ejaculatory disorders, dyspareunia, and infertility [45, 46, 47, 48, 49]. Although some of these problems are amenable to medicinal interventions, psychosocial issues contribute significantly to physical and psychological sexual dysfunction. They include change of status in the relationship, depression, anxiety, feelings of inadequacy, and body image issues [50, 51] that may arise due to the inability to return to employment. Survivors are not only less likely to be sexually active but also experience a reduction in the quality of sexual activity [50]. Although most male survivors are able to return to baseline sexual function after 2–3 years, the problems among female survivors persist even after long‐term follow‐up [46, 47, 48, 49].

Sexual dysfunction is a common complication following allo‐HSCT, yet it is frequently not adequately addressed. A study reported that only about 50% of the participants had discussions with their physicians regarding sexual concerns [52]. More than half of the women were interested in discussing the impact of treatment on sexual health, but 82% of HSCT recipients reported no such discussion with their providers [53]. Many survivors do attempt to address the issue of their sexuality but physicians often feel unqualified in this field, mainly because they view themselves to be insufficiently prepared to support their survivors.

## 4 A PROPOSED MULTIDISCIPLINARY MULTI‐PRONGED OCCUPATION‐FOCUSED APPROACH TO ENHANCE RETURNING TO EMPLOYMENT

Despite being widely accepted as a major problem faced by survivors following allo‐HSCT, only 36% of transplant centers in a survey reported having programs that address the problem of inability to return to employment [2]. Even so, information pertaining to the structure, design, and success of these programs is not available. Based on the discussion above, any effective return‐to‐employment program will need to incorporate solutions that address most, if not all, the long‐term psychosocial complications of survivors, in addition to working to address the survivors’ environment that may affect occupational performance, and evaluating the nature and demand of the survivors’ employment. Although for survivor‐related complications, medicinal approaches such as the use of anxiolytics, antidepressants, hormonal replacement therapy, and aggressive treatment of cGVHD are important components, many of the medications are associated with side effects. For example, medications for depression often induce sexual dysfunction [54, 55], and may not overcome the issue of fatigue and cognitive difficulties. The addition of complementary non‐medicinal approaches should, therefore, be considered. Since function deficits are the major problems faced by the survivors, it follows that an aggressive and innovative multidisciplinary multi‐pronged occupation‐focused approach that includes physicians, occupational therapists, physical therapists, psychologists, social workers, and employers may be the key to returning to employment. In this section, we propose such an approach and discuss the options available for specific complications.

### 4.1 A multidisciplinary multi‐pronged occupation‐focused approach

A multidisciplinary multi‐pronged occupation‐focused approach should aim to restore functionality, maintain, prevent, slow down deconditioning, and provide remediation or functional adaptation methods to the survivors. This approach may consist of two stages. The first stage aims to avoid deconditioning by providing early mobilization with work conditioning exercises [56] to prevent contractures, particularly in those affected by sclerotic chronic GVHD, and maintain or improve the range of movement, strength, endurance, and cognitive demands in work task simulations, such as engagement in simple tasks in alignment with the survivor's goal. For instance, for a survivor who hopes to return to employment working in an office, tasks that focus on cognitive and physical demands such as holding a telephone to have a conversation with their co‐workers, using computer keyboards, writing notes, and early functional mobility can be the short‐term goals. Following a work conditioning program, the second stage will be a work hardening program, which is an interdisciplinary, job‐specific program designed for the survivor. It uses real or simulated work tasks and progressive increase in the conditioning exercises, depending on the survivor's tolerances to maintain or improve the skills for a specific job the survivors performed previously, or to develop strategies to compensate for skills that cannot be restored by adjusting the work demands or the work environment to meet job demands and to promote independence at work.

In cases where the evaluation leads to a conclusion that the survivors will not be able to return to the same pre‐transplant job, vocational counseling may be suggested to enhance vocational skills for alternative job seeking and job acquisition.

Numerous occupation‐focused rehabilitation strategies have been applied to patients following therapy for various cancer types [57, 58, 59]. There is, however, a paucity of literature on strategies for survivors following allo‐HSCT. The functional strategies used for other cancer survivors may also be used on their own or in combinations for any specific complications following allo‐HSCT.

#### 4.1.1 Sleep disorders

Sleep disorders are often due to anxiety. One treatment approach for sleep includes Cognitive Behavioral Therapy (CBT) which examines distorted or negative thinking patterns and beliefs and restructures them to facilitate behavioral and emotional changes to benefit the survivors [60]. CBT can facilitate the identification of barriers that impact rest and relaxation and, based on these, develop an efficient sleeping hygiene program for the survivors. CBT has been used with benefits in randomized studies for sleep disorders in cancer survivors [61, 62, 63]. The importance of establishing a pre‐sleep routine that creates a consistent sleep‐wake pattern [64] should also be emphasized. These may include stretching, breathing techniques, meditation, and functional cognitive strategies. Simple measures such as avoiding caffeine in the afternoon or evening, avoiding exercise three hours before sleep, and using the bed only for sleeping or intimacy, should also be included.

#### 4.1.2 Anxiety/depression

Worry, fear, or loss of control impact/alter behavior and can establish a fear cycle that distorts one's perception of oneself [65]. While careful anxiolytics may alleviate the symptoms, the addition of complementary approaches from a multidisciplinary team is advocated. These interventions may include breathing exercises, meditation, progressive muscle relaxation, visualization techniques with arts and crafts, reading, and writing for self‐awareness, exploration, identification, and self‐expression, all of which can promote self‐efficacy and self‐confidence [66]. Group interventions that use a psychodynamic frame of reference (FOR) may allow a more profound connection to self through expressive art activities and the development of appropriate and healthy relationships between the members of the therapy/support group, allowing the survivors to identify stressors, develop coping techniques, and restore or promote intrinsic motivation while promoting mental and physical function. The psychodynamic FOR has been applied to cancer survivors with mixed results [67, 68, 69]. Its role in survivors following allo‐HSCT, however, remains to be determined.

Early identification and intervention of depression are vital. Simple routines and grading activities for success will promote self‐esteem and self‐efficacy. The addition of CBT may also help to uncover distorted beliefs and faulty thinking behaviors to redirect patients toward consistent, realistic, and healthy thinking patterns to improve self‐esteem, self‐confidence, self‐efficacy, and interpersonal skills [70] needed for successful return to employment.

#### 4.1.3 Cognitive impairment

An effective multidisciplinary multi‐pronged occupation‐focused therapy for this group of survivors with cognitive impairment will involve an assessment of the potential to minimize the level of impairment based on either the Cognitive Orientation to daily Occupational Performance (CO‐OP) model [71] or the Cognitive Disabilities (CD) model [72] so that the appropriate interventions may be applied. CO‐OP focuses on skill acquisition and uses problem‐solving for the transfer of learning, whereas the CD model presents routine tasks that a person can perform or that have been adapted to perform. A restorative approach can also be applied to remediable cognitive deficits, including interventions such as memory drills, block designs, and arranging blocks in a geometric pattern. Cognitive deficits due to comorbidities may be treated with repetitive practice of functional tasks. In survivors whose cognition is irreversibly damaged, occupation‐focused approaches that may be applied include compensatory strategies such as simple routines, checklists, electronic planners, reminders, simple written words to complete steps of tasks, or physical cues. These strategies, when applied appropriately, will assist the survivors in returning to employment.

#### 4.1.4 Fatigue

Many studies have demonstrated the variable benefits of exercise programs in chemotherapy‐induced fatigue (Table 1)[73, 74, 75, 76, 77, 78]. They include aerobic exercise, resistance exercise, and mindfulness exercise. Studies involving aerobic exercises produced mixed results on benefits [73, 74, 75, 76], while the beneficial effects of resistance exercise have been more consistent [77, 78]. Management of fatigue may also involve educating survivors on strategies to promote quality of movement while using energy wisely. Survivors may be taught to minimize long static holding postures, take frequent breaks, alternate sitting and standing during functional activities, and avoid unnecessary repetition or trips. It is highly recommended to keep a diary throughout the day to monitor activity levels and identify occupations and activities that result in fatigue. If the survivor is unable to restore function or the activity is very demanding, compensatory strategies may be used, such as grading down the activity, adjusting to the environment, or using assistive devices to assist with functional independence. Education on body mechanics and positioning, and strategies such as planning ahead, prioritizing, breaking down tasks into components, task simplification, and adding resting periods when needed will assist survivors to perform meaningful personal needs safely. In addition to planning short rest periods between functional components of tasks or occupations, survivors may progress to balance light work tasks with heavy tasks. Strategies that can be used along with breaks are organization and gathering of all the items and equipment necessary prior to the activities and use of assistive devices such as a cart, bucket, or backpack to carry items to avoid multiple trips. Non‐essential tasks should be eliminated until the survivor is able to manage them independently. Survivors who are unable to perform some tasks can delegate them or combine them using props such as high stools, avoiding bending and stooping, sliding rather than lifting heavy items, using light items, and taking intermittent rest breaks rather than resting after exhaustion. Improvement of symptoms of fatigue increases the likelihood of readiness to return to employment.

**TABLE 1:** Results of a sample of studies on exercise in allogeneic hematopoietic stem cell transplant (allo‐HSCT)‐associated fatigue.

Study	Study design	Sample size (*n*)	Intervention	Results	Conclusions
Jarden et al. [73]	Randomized	*n* = 34	Aerobic exercise and resistance training	Improvement in both QoL, fatigue, and mental health but did not reach statistical significance	Exercise in patients undergoing allo‐HSCT did not have adverse effects but did not improve symptoms of fatigue significantly
Shelton et al. [74]	Randomized	*n* = 53	Aerobics and resistance training three times a week for 4 weeks	Fatigue levels decreased in both groups after 4 weeks, but the difference did not reach any statistically significant	Exercise did not produce any statistically significant difference compared to the control group
Baumann et al. [75]	Randomized	*n* = 33	Exercise group: Aerobic exercise with daily activities twice a day. Control group: Standard clinical physiotherapy once a day	Improvement in fatigue in the exercise group (*p* = 0.046)	Exercise improved physical function and quality of life following allo‐HSCT
Knols et al. [76]	Randomized	*n* = 114	Aerobic exercise and resistance training twice a week for 12 weeks	The fatigue score improvements trended toward statistical significance (*p* = 0.056)	Exercise may be effective in the improvement of physical function after hospital discharge following allo‐HSCT
Wiskemann et al. [77]	Randomized	*n* = 80	Three times a week, two sessions of resistance training once a week before admission for transplant, two sessions of resistance training during hospital stay, and five sessions of exercise once a week after discharge	Exercise significantly improved fatigue scores (*p* < 0.01–0.03), physical fitness/functioning (*p* = 0.02–0.03), and global distress (*p* = 0.03).	Exercise reduced fatigue and stress and enhanced physical performance and QoL after allo‐HSCT
Bargi et al. [78]	Randomized	*n* = 38	Inspiratory muscle training for 6 weeks	Improvement in fatigue scores (*p* < 0.05)	Inspiratory muscle training was effective in improving respiratory muscle strength, exercise capacity, and breathing, and reducing depression and fatigue

Allo‐HSCT = allogeneic hematopoietic stem cell transplant; QoL = Quality of life.

#### 4.1.5 Sexual dysfunction

An approach commonly employed when addressing sexual dysfunction is the Permission, Limited Information, Specific Suggestions, and Intensive Therapy (PLISSIT) model [79]. PLISSIT is a model in sexology to determine the different levels of intervention for individual survivors. Early use of PLISSIT may prepare survivors for the expected sexual impacts and begin the process of restoring sexual function. Sexual rehabilitation interventions include modification techniques for sexual expression such as hugging, kissing, foreplay, and other meaningful areas using gradation, modification, simplification, or alteration of the goal, in addition to vaginal dilation and local estrogen therapy, especially for those affected by genital cGVHD [80, 81] following allo‐HSCT. Sexual education for survivors and their loved ones can provide effective therapeutic interventions to manage unexpected or undesirable reactions or behaviors, such as demeaning or resentment that affect the survivor's dignity and esteem.

### 4.2 Returning to employment

The complication of inability to return to full‐time employment is most acute during the first year after transplant because, in addition to the psychosocial issues, the problem is also compounded by the immunosuppression, increased risks for infections, and symptoms due to GVHD and its treatment. Although financial concerns may be a major driver to survivors wanting to return to employment, this is, however, only possible if the survivors are equipped with the appropriate physical and mental abilities needed for satisfactory occupational performance. A multidisciplinary occupation‐focused approach is, therefore, important, and should start from Day 1 of hospital admission for allo‐HSCT. Every effort should also be made to tailor the transplant types and preparative regimens to be consistent with the patient's desire to return to employment and the nature of his/her employment. Occupational rehabilitation should be proactive rather than reactive. Once the survivors’ basic goals such as Activities of Daily Living (ADL) are attained, interventions should proceed to address survivors' goals that are more demanding in complexity such as Instrumental ADL or work. Strategies to help return to employment may include creating, producing, and distributing products and services, managing time, relationships with the team and customers, and complying with norms and procedures. To progress from basic ADL to more complex occupations, survivors must demonstrate the physical, cognitive, and psychosocial skills required to replicate the actual demands of the job. An occupation‐focused approach may provide the survivors with context or task modification and the use of assistive devices to facilitate performance. It may also help to identify existing limitations and barriers before returning to work and advocate for survivors' accommodations when needed to promote safety, independence, and QoL. This may particularly be helpful in survivors with sclerotic cGVHD or those affected by musculoskeletal involvement of the disease. The biomechanical FOR aims to prevent deterioration or maintain existing abilities or to alleviate disabilities with technology or equipment as a form of compensatory intervention. It is not uncommon to use both biomechanical and rehabilitation FOR at any point of the occupation‐focused interventions regardless of the nature of survivors' characteristics, stage of the disease, or demands of the activity.

It should be noted that addressing the physical and psychosocial complications of survivors is only one of many components needed for the successful return to employment. Active social work involvement in collaboration between the multidisciplinary team and the employer should explore the nature of the survivor's pre‐transplant employment and his/her work environment, as proposed by the PEO model and complemented by the pre‐emptive use of the Feuerstein approach which involves dynamic assessment, cognitive activation, mediated learning and shaping a modifying environment [82]. There is a need to consider the survivor's health and functional status in relation to his/her work demands, work environment, workplace policy and procedures, and financial incentives to the survivors so that potential work problems can be conceptualized and the appropriate adaptation may be made. Other approaches may involve working remotely, although this may not be always possible, depending on the nature of the employment.

## 5 CONCLUSION

Despite being a major problem facing survivors following allo‐HSCT, the inability to return to employment is often not discussed with survivors, probably a reflection that most physicians do not feel equipped to address the issue [83, 84]. Employers also usually do not understand fully how cancer might impact their employees and their work [85]. This is further complicated by the multitude of long‐term challenges the survivors face.

Although the reasons for the inability to return to employment are complex and the consequences of physical and psychosocial problems affecting survivors following allo‐HSCT are significant, functional therapeutic interventions appear to have an important role in helping to alleviate this complication. A study of 84 hospital staff including nurses, physicians, occupational therapists, psychologists, social workers, physical therapists, and dietitians in three transplant centers in the United Kingdom found that psychosocial supportive care services were variable and mostly reactive rather than pro‐active [86], highlighting a lack of a multidisciplinary involvement early and routinely in the course of treatment. Since many of the challenges are functional in nature, it follows that early occupation‐focused interventions may be key to success. Understanding the survivor's home and employment setup, together with his/her social support will enable the design of appropriate return‐to‐employment plans according to the survivor's needs and goals for the post‐transplant period. Therefore, instead of being reactive and waiting until the survivors are deconditioned and require intervention for function, early occupation‐focused interventions aim to maintain and minimize deterioration of the pre‐transplant physical and psychological well‐being. This, however, needs to be done in collaboration with the transplant physicians and the therapists, rather than in isolation as is commonly the case, to set expectations that are agreed upon between the survivors, physicians, and therapists. Education on issues of and obstacles to returning to employment should be provided to the survivors, partners/spouses, and families prior to allo‐HSCT.

It should, however, be noted that finding fulfillment and contentment in life is not just confined to PEO. Some patients may be content with being unemployed and being able to spend more time with their families and their loved ones, due to a change in their perspective on lives after surviving cancer. Further work is needed to identify the prevalence of this group of patients who may contribute to the apparent higher rates of unemployment among survivors.

In summary, the inability to return to pre‐transplant employment continues to be a major problem following allo‐HSCT. A proactive multidisciplinary multi‐pronged approach that involves a close working relationship between the transplant physicians, nurses, occupational therapists, psychologists, social workers, and employers early during allo‐HSCT is much needed. More aggressive GVHD prophylaxis and therapy may also alleviate the prevalence of inability to return to employment in these survivors. Finally, it must be appreciated that the role of peer support groups and family/caregiver support is as important. Until all these are accomplished and novel multidisciplinary multi‐pronged approaches are used, the QoL of survivors following allo‐HSCT will continue to be compromised.

## AUTHOR CONTRIBUTIONS

Conceptualization: SHL and LF; methodology: SHL; validation, SHL and RM; formal analysis: SHL; investigation: LF and AS; writing—original draft preparation: LF, AS, RM; writing—review and editing and final draft: SHL. All authors have read and agreed to the published version of the manuscript.

## CONFLICT OF INTEREST STATEMENT

The authors declare no conflict of interest.

## FUNDING INFORMATION

None

## ETHICS STATEMENT

The authors have confirmed ethical approval statement is not needed for this submission.

## CLINICAL TRIAL REGISTRATION

The authors have confirmed clinical trial registration is not needed for this submission.

## PATIENT CONSENT STATEMENT

The authors have confirmed patient consent statement is not needed for this submission.

## Data Availability

Not applicable.
